# Phase Diagram of Binary Alloy Nanoparticles under High Pressure

**DOI:** 10.3390/ma14112929

**Published:** 2021-05-29

**Authors:** Han Gyeol Kim, Joonho Lee, Guy Makov

**Affiliations:** 1 Department of Materials Science and Engineering, Korea University, Seoul 02841, Korea; pureblue7@korea.ac.kr; 2 Department of Materials Engineering, Ben-Gurion University of the Negev, Beer-Sheva 84105, Israel

**Keywords:** CALPHAD, nanoparticles, pressure, phase diagrams, eutectic point

## Abstract

CALPHAD (CALculation of PHAse Diagram) is a useful tool to construct phase diagrams of various materials under different thermodynamic conditions. Researchers have extended the use of the CALPHAD method to nanophase diagrams and pressure phase diagrams. In this study, the phase diagram of an arbitrary A–B nanoparticle system under pressure was investigated. The effects of the interaction parameter and excess volume were investigated with increasing pressure. The eutectic temperature was found to decrease in most cases, except when the interaction parameter in the liquid was zero and that in the solid was positive, while the excess volume parameter of the liquid was positive. Under these conditions, the eutectic temperature increased with increasing pressure.

## 1. Introduction

During the last several decades, computational modeling of phase diagrams using the CALPHAD (CALculation of PHAse Diagrams) method has been applied to various systems under extreme conditions of small particles and high pressures [1,2,3,4,5,6,7,8,9,10,11,12,13,14,15,16,17,18,19,20,21,22,23,24,25,26,27,28,29,30,31,32,33,34,35,36,37,38,39,40,41,42,43,44,45,46,47,48,49,50,51,52,53,54,55,56,57,58,59,60,61,62]. Weissmüller first suggested a simple CALPHAD-type thermodynamic model of nanostructures [1,2]. Later, Tanaka et al. incorporated the Butler equation into the thermodynamic modeling of nanoparticles to evaluate the surface effect [3,4,5]. Park and Lee proposed CALPHAD-type thermodynamic equations, which can be used directly in commercial thermodynamic software [6,7]. This method has been extended to nanowire and nanofilm systems [8]. Recently, Sim and Lee successfully assessed the phase diagrams of nanoparticles containing intermetallic compounds [9]. Application of this model to pure metallic nanoparticles has been validated experimentally and theoretically [10,11,12,13,14,15,16,17,18,19]. The validity of this model has also been confirmed through phase diagram studies of various alloy nanoparticle systems (Ag-Au [6,8,20,21,22], Ag-Co [23], Ag-Cu [24,25,26], Ag-Sn [9,27,28,29,30], Au-Cu [31], Au-Pt [32], Bi-Cd [33], Bi-Sn [34,35], Cu-Ni [36,37,38,39,40,41], Cu-Pt [42], In-Sb [43], Ni-Sn [44], Si-Ge [45,46], etc.). This model has been extended to phase diagram studies on ceramic nanoparticles [47,48,49,50] and aerosol nanoparticles [51]. Studies of nanowires in the frame of the CALPHAD method can be found in the literature [52,53,54,55]. On the other hand, phase diagrams of various materials under high pressure have also been assessed using the framework of the CALPHAD method [56,57,58,59]. Ben Shalom et al. reported the pressure diagram of the Ga-In system calculated with bulk thermodynamic data and highly accurate sound velocity and density data [56]. Similar works have been carried out for various systems, such as Bi-Sn [57,58], Bi-Sb [58,59], and Pb-Sn [58,59]. These studies demonstrate that this methodology can be applied to systems with high pressures of up to several GPa. Although obtaining the phase diagram is essential when designing new materials and processes, experimental determination of the phase diagram under extreme conditions remains challenging [60].

On the other hand, several researchers have reported on the phase stability of nanoparticles under high pressure, which differs from that of bulk materials under ambient pressure. Tolbert and Alivisatos investigated the size dependence of the phase transition of CdSe nanoparticles under high pressure [61]. They reported that the phase transformation pressure from wurtzite and zinc blende to rock-salt structure changed from 2.5–3.0 GPa for bulk material to 6.3 GPa for 4.4 nm nanoparticles. Qadri et al. reported that the transition pressure of PbS nanoparticles increases with a decrease in particle size [62]. Wang et al. found that the phase transition of 30 nm TiO_2_ nanoparticles from the anatase and rutile mixture to baddeleyite structure occurs at 8.7 GPa, whereas bulk TiO_2_ at 2–5 GPa transitions from anatase to columbite α-PbO_2_-type structure and a successive phase transformation to baddeleyite structure occurs at 12–15 GPa [63]. Daou et al. synthesized Fe_3_O_4_ nanoparticles using a hydrothermal process in which high pressure was applied at elevated temperatures [64]. Hsu et al. synthesized wurtzite-ZnO nanoparticles under pressures as high as 2 GPa [65]. In order to understand the phase stability of nanoparticles at high pressure and high temperature, the phase diagram of nanoparticles should be established at high pressure. However, due to the experimental difficulties of performing phase stability measurements under extreme conditions, it is not easy to establish a thermodynamic database for such phase diagram calculations. Nevertheless, it would be helpful to examine the effects of changes in thermodynamic and thermophysical properties on the phase stability of nanosystems under high pressure based on a simplified system. 

In this study, the phase diagram of spherical nanoparticles under high pressure is examined using a simplified arbitrary A–B binary regular solution model, which was suggested by Lee et al. [7]. Those authors investigated the effects of the interaction parameter on the phase stability of binary alloy nanoparticle systems using the simplified regular solution model. They found that the shape of the phase diagram significantly changed as the particle size decreased when the solid interaction parameter was positive and the liquid interaction parameter was less than or equal to zero, demonstrating a eutectic point. When the particle size decreased, the eutectic temperature slightly decreased, and the eutectic composition moved in the direction of the pure substance with a lower melting point. In the other cases, the melting points and liquidus temperatures of nanoparticles decreased continuously when the particle size decreased across the entire composition range. Recently, this regular solution model was also used to examine the effects of the interaction parameter and the excess volume on the pressure phase diagram [66]. Here, it was considered that positive and negative excess volumes corresponded to an increase and decrease in the interaction parameters, respectively. Therefore, in this study, attention is paid to binary alloy nanoparticle systems with a eutectic point. The effects of size and pressure were investigated while controlling the interaction parameter and the excess volume. In a real case, it is necessary to consider phase separation in a nanoparticle, as the presence of a solid–solid or solid–liquid interface may affect the phase stability of the nanoparticle. Thus, the present model is only validated when the two phases have the same curvature and share a facet interface. This assumption may not alter the direction of changes in the phase diagram, but the extent to which that holds true is not known [7].

## 2. Theory and Model

### 2.1. Effect of Nanoparticle Size 

As the size of a system decreases, the relative contribution of the surface (or interface) to its thermodynamic properties increases. Accordingly, the internal energy (*U*), enthalpy (*H*), Helmholtz energy (*A*), and Gibbs energy (*G*) can be described by Equations (1)–(4).

(1) $$dU=TdS-PdV+\underset{i}{\sum }\mu_{i}dn_{i}+\sigma ds$$

(2) $$dH=TdS+VdP+\underset{i}{\sum }\mu_{i}dn_{i}+\sigma ds$$

(3) $$dA=-SdT-PdV+\underset{i}{\sum }\mu_{i}dn_{i}+\sigma ds$$

(4) $$dG=-SdT+VdP+\underset{i}{\sum }\mu_{i}dn_{i}+\sigma ds$$

where *S* is the entropy, *T* is the temperature, *P* is the pressure, *V* is the volume, *n_i_* is the number of moles of *i*, *μ_i_* is the chemical potential of *i*, *s* is the surface area, and *σ* is the surface tension. In this paper, it is simply assumed that the contribution of interfacial energy to thermodynamic properties is negligible. From the definition, the chemical potential of *i* can be described by Equation (5).

(5) $$\mu_{i}={\left(\frac{\partial U}{\partial n_{i}}\right)}_{S,V,n_{j},s}={\left(\frac{\partial H}{\partial n_{i}}\right)}_{S,P,n_{j},s}={\left(\frac{\partial A}{\partial n_{i}}\right)}_{T,V,n_{j},s}={\left(\frac{\partial G}{\partial n_{i}}\right)}_{T,P,n_{j},s}$$

It is difficult to fix the entropy and volume for a nanoparticle system. Therefore, the chemical potential is generally defined from the Gibbs energy. Here, it should be noted that all of the variables are independent. However, this hypothesis is not valid in a nanoparticle system because the surface area (*s*) is affected by the change in mole number (*n_i_*). Thus, an infinitesimal change in the surface area (*ds*) should be suggested by a function of the mole number (*dn_i_*) [8]. For simplicity of calculation, a spherical nanoparticle is assumed. For an isotropic spherical particle, the volume and the surface of the particle can be described with respect to the particle’s radius (*r*) by Equations (6) and (7), respectively.

(6) $$V=\frac{4}{3}\pi r^{3}$$

(7) $$s=4\pi r^{2}=\frac{3V}{r}$$

Differentiating Equations (6) and (7) with respect to *r*, the volume and the surface changes are given by Equations (8) and (9), respectively.

(8) $$dV=4\pi r^{2}dr$$

(9) ds=8πrdr

Incorporating Equations (8) and (9), *ds* can be approximated as a function of *dn_i_* (Equation (10)).

(10) $$ds=\frac{2}{r}dV=\frac{2}{r}\sum V_{i}dn_{i}$$

Then, Equation (4) is replaced by Equation (11).

(11) $$dG=-SdT+VdP+\underset{i}{\sum }\left(\mu_{i}+\frac{2\sigma V_{i}}{r}\right)dn_{i}$$

Consequently, the chemical potential of *i* of a nanoparticle system is expressed by Equation (12) [8].

(12) $$\mu_{i}^{NP}={\left(\frac{\partial G}{\partial n_{i}}\right)}_{T,P,n_{j}}=\mu_{i}^{Bulk}+\frac{2\sigma V_{i}}{r}$$

When it is assumed instead that the surface area (*s*) and the mole number (*n_i_*) are independent variables, the second term of Equation (12) can be written as 3*σV_i_*/*r* [8]. 

For simplicity, let us consider an A–B binary alloy nanoparticle system. It is also assumed that the nanoparticle is isotropic spherical. Then, the molar Gibbs energy of the nanoparticle system is expressed by Equation (13) [3,4].

(13) $$G_{m}^{NP}=G_{m}+G^{NP,\;Surf.}$$

where $G_{m}$ and $G^{NP,\;Surf.}$ are the contributions of the bulk and the surface to the molar Gibbs energy, respectively. From the analogy of bulk thermodynamic description, Equation (13) can be described by Equation (14) [8].

(14) $$G_{m}^{NP}=X_{A}G_{A}^{oNP}+X_{B}G_{B}^{oNP}+RT\left(X_{A}lnX_{A}+X_{B}lnX_{B}\right)+G^{Ex,NP}$$

where $X_{i}$ is the mole fraction of *i* (=*A*, *B*), $G_{i}^{oNP}$ is the standard molar Gibbs energy of the nanoparticle of pure *i*, *R* is the gas constant, *T* is the temperature and $G^{Ex,NP}$ is the excess Gibbs energy of the nanoparticle system [8].

(15) $$G_{i}^{oNP}=G_{i}^{o}+\frac{2\sigma_{i}V_{i}}{r}$$

(16) $$G^{Ex,NP}=G_{m}+\frac{2\sigma V}{r}-\left(X_{A}G_{A}^{oNP}+X_{B}G_{B}^{oNP}+RT\left(X_{A}lnX_{A}+X_{B}lnX_{B}\right)\right)$$

where σ is the surface tension of the alloy, *V* is the molar volume of the alloy, *r* is the radius of the nanoparticle, $\sigma_{i}$ is the surface tension of pure *i*, and $V_{i}$ is the molar volume of pure *i*. Assuming a regular solution, the excess Gibbs energy of the nanoparticle system can be simplified to Equation (17) using the interaction parameter $\Omega^{NP}$ [1,2,19].

(17) $$G^{Ex,NP}=\Omega^{NP}X_{A}X_{B}=X_{A}X_{B}\sum L_{k}{}^{NP}{\left(X_{A}-X_{B}\right)}^{k}$$

where $L_{k}{}^{NP}\left(k=0,\;1,\;2,\ldots \right)$ is the Redlich–Kister constant of the nanoparticle system. 

The surface tension of the A–B alloy is calculated by Butler’s equation.

(18) $$\sigma =\sigma_{A}+\frac{RT}{A_{A}}\ln\left(\frac{X_{A}^{s}}{X_{A}^{b}}\right)+\frac{\Omega^{Bulk}}{A_{A}}\left(\beta {\left(X_{B}^{s}\right)}^{2}-{\left(X_{B}^{b}\right)}^{2}\right)\;=\sigma_{B}+\frac{RT}{A_{B}}\ln\left(\frac{X_{B}^{s}}{X_{B}^{b}}\right)+\frac{\Omega^{Bulk}}{A_{B}}\left(\beta {\left(X_{A}^{s}\right)}^{2}-{\left(X_{A}^{b}\right)}^{2}\right)$$

where $\sigma_{i}$ is the surface tension of a pure element, $A_{i}$ is the molar surface area of pure *i* ($A_{i}=1.091N_{A}{}^{1/3}V_{i}{}^{2/3}$ where $N_{A}$ is Avogadro’s number and $V_{i}$ is the molar volume of pure *i*), $X_{i}^{s}$ is the surface composition of *i*, $X_{i}^{b}$ is the bulk composition of *i*, $\Omega^{Bulk}$ is the bulk interaction parameter, and β is the parameter corresponding to the ratio of the coordination number in the surface to that in bulk. Park and Lee showed that the values of β for liquid metals and solid metals are 0.85 and 0.84, respectively [6]. 

The molar volume of the alloy is calculated by Equation (19).

(19) $$V=X_{A}V_{A}+X_{B}V_{B}+V^{Ex}=X_{A}V_{A}+X_{B}V_{B}+V_{A,B}X_{A}X_{B}$$

where $V^{Ex}$ is the excess volume and $V_{A,B}$ is the excess volume parameter. In this study, it is assumed that $V_{A,B}$ for the solid is zero, because the excess volume of a liquid is generally much larger than that of the corresponding solid. 

### 2.2. Effect of Pressure on Thermodynamic Equations

The molar Gibbs energy of a system under high pressure, $G_{m}^{P}$, is expressed by Equation (20):

(20) $$G_{m}^{P}=G_{m}+{\left(\frac{\partial G}{\partial P}\right)}_{P\to 0}P=G_{m}+VP$$

where $G_{m}$ is the molar Gibbs energy of pure *i* under atmospheric pressure, *V* is the molar volume of a solution, and *P* is the pressure. 

On the other hand, the molar Gibbs energy of a system under high pressure can be rewritten by Equation (21).

(21) $$G_{m}^{P}=X_{A}G_{A}^{P}+X_{B}G_{B}^{P}+RT\left(X_{A}lnX_{A}+X_{B}lnX_{B}\right)+G^{Ex,P}$$

where $G_{i}^{P}$ is the molar Gibbs energy of pure *i* under pressure *P* and $G^{Ex,P}$ is the excess Gibbs energy under pressure *P*. $G_{i}^{P}$ and $G^{Ex,P}$ are given by Equations (22) and (23), respectively.

(22) $$G_{i}^{P}=G_{i}^{o}+{\left(\frac{\partial G_{i}}{\partial P}\right)}_{P\to 0}P=G_{i}^{o}+V_{i}P$$

where $G_{i}^{o}$ is the standard molar Gibbs energy of pure *i* and $V_{i}$ is the molar volume of pure *i* under atmospheric pressure.

(23) $$G^{Ex,P}=G^{Ex}+{\left(\frac{\partial G^{Ex}}{\partial P}\right)}_{P\to 0}P=G^{Ex}+V^{Ex}P$$

where $G^{Ex}$ is the bulk excess Gibbs energy under atmospheric pressure and $V^{Ex}$ is the excess volume, which can be expressed by Equation (24).

(24) $$V^{Ex}=V-V_{ideal}=V-\left(X_{A}V_{A}+X_{B}V_{B}\right)=\frac{M}{\rho }-\left(X_{A}\frac{M_{A}}{\rho_{A}}+X_{B}\frac{M_{B}}{\rho_{B}}\right)\;={\left(\frac{\partial G^{Ex}}{\partial P}\right)}_{p\to 0}=X_{A}X_{B}\sum {\left(\frac{\partial L_{k}{}^{P}}{\partial P}\right)}_{p\to 0}{\left(X_{A}-X_{B}\right)}^{k}$$

where $V_{ideal}$ is the hypothetical volume of the alloy by ideal mixing, M is the molar mass of the alloy, ρ is the density of the alloy, $M_{i}$ is the molar mass of pure *i*, and $\rho_{i}$ is the density of pure *i* [56,57,58,59]. Here, it is assumed that the excess volume is constant at high pressures up to several GPa.

Finally, the excess Gibbs energy of a system under high pressure can be simplified to Equation (25) using the interaction parameter under high pressure $\Omega^{P}$.

(25) $$G^{Ex,P}=G^{Ex}+V^{Ex}P=\Omega^{P}X_{A}X_{B}=X_{A}X_{B}\sum L_{k}{}^{P}{\left(X_{A}-X_{B}\right)}^{k}$$

### 2.3. Thermodynamic Equations of Nanoparticles under High Pressure 

Now, the effects of particle size and pressure can be merged into Equation (26).

(26) $$G_{m}^{NP,\;P}=X_{A}G_{A}^{oNP,P}+X_{B}G_{B}^{oNP,P}+RT\left(X_{A}lnX_{A}+X_{B}lnX_{B}\right)+G^{Ex,NP,P}$$

The standard molar Gibbs energy of the nanoparticle of pure *i* under pressure *P* is expressed by Equation (27).

(27) $$G_{i}^{oNP,P}=G_{i}^{o}+\frac{2\sigma_{i}V_{i}}{r}+V_{i}P$$

The excess Gibbs energy is expressed by Equation (28).

(28) $$G^{Ex,NP,P}=G^{Ex,NP}+V^{Ex}P=X_{A}X_{B}\sum L_{k}{}^{NP,P}{\left(X_{A}-X_{B}\right)}^{k}$$

### 2.4. Phase Equilibria Model

In this study, a regular solution model for arbitrary A–B systems with a eutectic point is considered. The melting points of the end-members, A and B, are assumed to be 1200 and 800 K, respectively. When pure solid *i* is taken as the standard state (assuming that $G_{i\left(s\right)}^{o}=0$), the Gibbs energy of pure liquid *i* is assumed to be expressed by Equation (29) [7].

(29) $$G_{i\left(L\right)}^{o}=S_{m,i}\left(T_{m,i}-T\right)$$

where the melting point entropy of pure *i*, $S_{m,i}$, is assumed to be 8.8 J K^−1^mol^−1^ according to Richard’s rule [67]. Here, $T_{m,i}$ is the melting point of pure *i*.

A systematic study by Lee et al. on the shape of phase diagrams of a simple binary alloy system reported that a eutectic point was observed when the excess Gibbs energy of a liquid was zero or negative while the excess Gibbs energy of the solid was positive [7]. Therefore, in the present study, $G_{\left(L\right)}^{Ex}$ is assumed to be $-15,000X_{A}X_{B}$ and 0 J mol^−1^, while $G_{\left(s\right)}^{Ex}$ is $15,000X_{A}X_{B}$ J mol^−1^. 

The surface tensions of the pure liquid and solid are approximated by Equations (30) and (31), respectively [7].

(30) $$\sigma_{i\left(l\right)}=0.00075T_{m,i}-0.0001\left(T-T_{m,i}\right)\;(N\;m^{-1})$$

(31) $$\sigma_{i\left(s\right)}=0.00087T_{m,i}-0.0001\left(T-T_{m,i}\right)\;(N\;m^{-1})$$

Assuming 4% volume expansion upon melting and a temperature coefficient of 10^−4^ K^−1^, the molar volume of the pure solid and liquid can be expressed by Equations (32) and (33), respectively [7].

(32) $$V_{i\left(s\right)}=1.00\times 10^{-5}\;(m^{3}\;{\mathrm{mol}}^{-1})$$

(33) $$V_{i\left(l\right)}=1.04\times 10^{-5}\times \left\{1+10^{-4}\left(T-T_{i,m}\right)\right\}\;(m^{3}\;{\mathrm{mol}}^{-1})$$

The excess volume parameter of the solid is assumed to be zero ($V_{A,B\left(s\right)}=0$), whereas that of the liquid ($V_{A,B\left(l\right)}$) is −10^−6^, 0, and 10^−6^ m^3^ mol^−1^.

## 3. Results and Discussion

### 3.1. Variations in the Shape of the Phase Diagram

**Figure 1:**
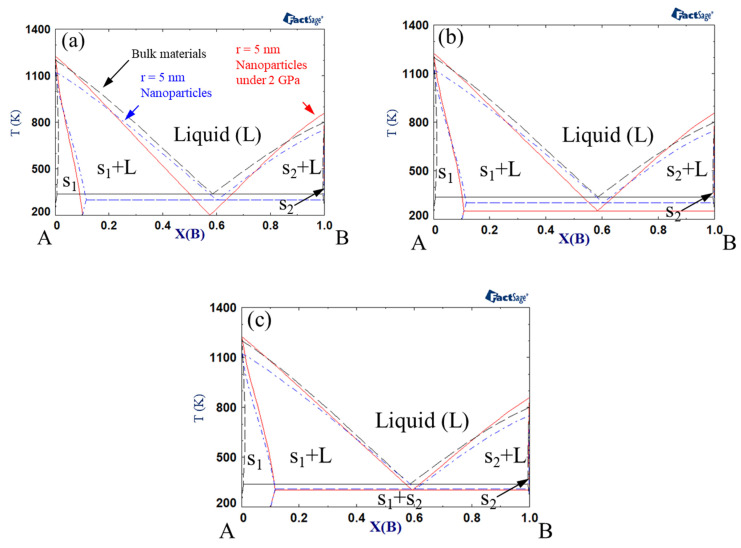
A–B phase diagram of each case (black dashed line indicates bulk material; blue dash-dotted line indicates 5 nm radius nanoparticles, red solid line indicates 5 nm radius nanoparticles under 2 GPa): Ω*_s_* = 15 kJ/mol, Ω*_L_* = −15 kJ/mol, (**a**) *V^Ex^* = −1 cm^3^/mol, (**b**) *V^Ex^* = 0 cm/mol, (**c**) *V^Ex^* = 1 cm^3^/mol.

Phase diagrams were calculated by Gibbs energy minimization with FactSage software [68,69]. Figure 1 shows the phase diagrams of bulk material, nanoparticles, and nanoparticles under a pressure of 2 GPa. Here, the interaction parameter of bulk solid under atmospheric pressure was +15 kJ mol^−1^, whereas that of bulk liquid under atmospheric pressure was −15 kJ mol^−1^. The interaction parameter could be modified by a size effect or pressure effect. The size of each nanoparticle was assumed to be *r* = 5 nm. The excess volume parameter for liquid ($V_{A,B\left(l\right)}$) varied between −10^−6^, 0, and +10^−6^ m^3^ mol^−1^. Notably, the melting points of the pure substances (A and B) decreased for nanoparticles vs. bulk material, while it increased when high pressure was applied to the nanoparticles. On the other hand, the eutectic point decreased for nanoparticles, and it decreased further when high pressure was applied to the system. The extent of the reduction in the eutectic temperature became greater when the excess volume parameter was negative. Makov et al. demonstrated that the eutectic temperature of bulk binary alloys decreases as the solid interaction parameter increases or the liquid interaction parameter decreases [66]. The same authors also found that the effects of positive and negative excess volumes at high pressure correspond to the increase and decrease in the interaction parameters, respectively. Since the excess volume of the solid is negligible compared to that of the liquid, a negative excess volume of liquid in the present study results in the same effect as a decrease in the liquid interaction parameter. Consequently, the eutectic temperature of the nanoparticles decreased with increasing pressure. 

**Figure 2:**
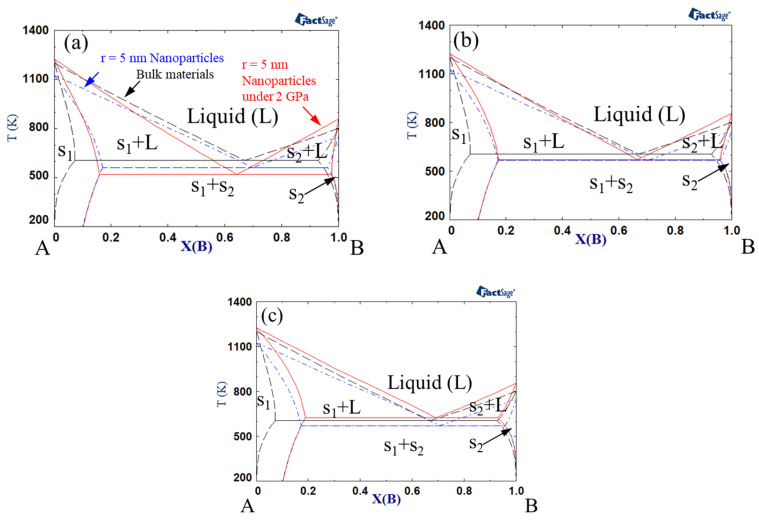
A–B phase diagram of each case (black dashed line indicates bulk material, blue dash-dotted line indicates 5 nm radius nanoparticles, red solid line indicates 5 nm radius nanoparticles under 2 GPa): Ω*_s_* = 15 kJ/mol, Ω*_L_* = 0 kJ/mol, (**a**) *V^Ex^* = −1 cm^3^/mol, (**b**) *V^Ex^* = 0 cm^3^/mol, (**c**) *V^Ex^* = 1 cm^3^/mol.

Figure 2 shows the phase diagrams when the interaction parameter of the solid was +15 kJ mol^−1^, while that of the liquid was 0 kJ mol^−1^. The eutectic temperature generally increased in comparison to the calculation results shown in Figure 1. When the excess volume parameter for liquid was −10^−6^ m^3^ mol^−1^, the eutectic temperature decreased with the change from bulk to nanoparticles and nanoparticles under high pressure. However, when the excess volume parameter of the liquid was +10^−6^ m^3^ mol^−1^, the eutectic temperature decreased with the change from bulk to nanoparticles, whereas it increased when high pressure was applied to the nanoparticles. As described above, an increase in the liquid interaction parameter from −15 kJ mol^−1^ to 0 kJ mol^−1^ yielded an increase in eutectic temperature. 

### 3.2. Variations in the Eutectic Points

**Figure 3:**
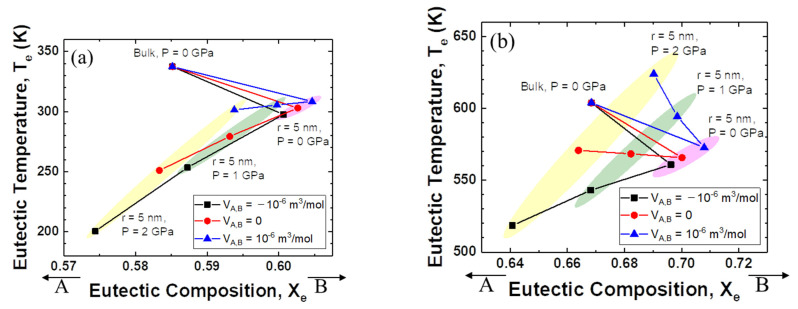
Eutectic temperature and eutectic composition of A–B phase diagram of each case (negative excess Gibbs energy parameter of liquid/zero excess Gibbs energy parameter of liquid): (**a**) Ω*_s_* = 15 kJ/mol, Ω*_L_* = −15 kJ/mol, (**b**) Ω*_s_* = 15 kJ/mol, Ω*_L_* = 0 kJ/mol.

Figure 3a shows the relationship between eutectic composition and eutectic temperature when the interaction parameter of the solid is +15 kJ mol^−1^ and that of the liquid is −15 kJ mol^−1^. For the change from bulk to nanoparticles, the eutectic composition moves to the direction of the low melting temperature element, B. When high pressure is applied, the eutectic composition moves back in the direction of the high melting temperature element, A. The changes in temperature and composition are more significant when the excess volume parameter of the liquid is negative. 

Figure 3b shows more interesting results. The direction of movement of the eutectic composition is the same as shown in Figure 3a. However, the change in the eutectic temperature depends on the sign of the excess volume parameter for liquid. When the excess volume parameter has a negative value, the eutectic temperature decreases for the change from bulk to nanoparticles, and for the change from atmospheric pressure to high pressure. On the other hand, when the excess volume parameter is zero, the eutectic temperature does not change much as the pressure increases. Surprisingly, when the excess volume parameter is positive, the eutectic temperature rises rapidly with increasing applied pressure. 

**Figure 4:**
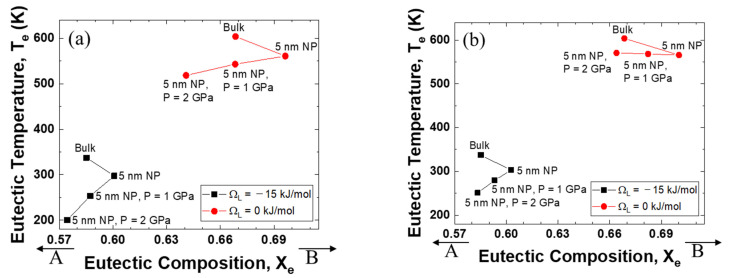

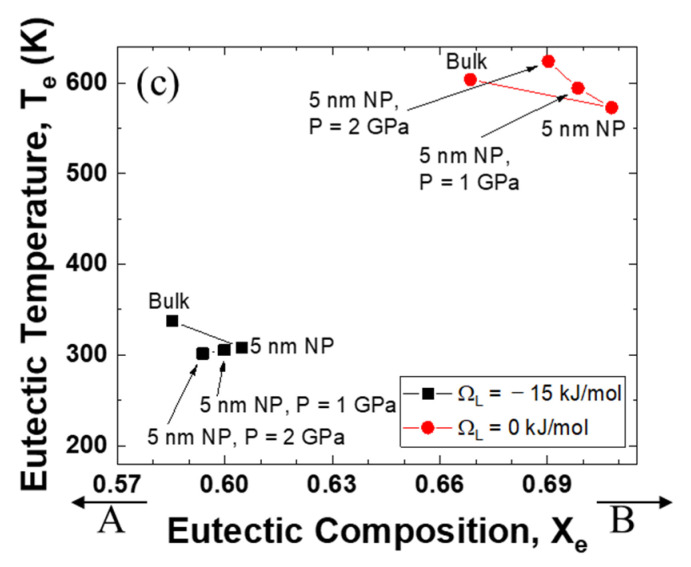
Eutectic temperature and eutectic composition of A–B phase diagram of each case (negative excess volume parameter of liquid/zero excess volume parameter of liquid/positive excess volume parameter of liquid): (**a**) *V^Ex^* = −1 cm^3^/mol, (**b**) *V^Ex^* = 0 cm^3^/mol, (**c**) *V^Ex^* = 1 cm^3^/mol.

Figure 4 illustrates the relationship between the eutectic composition and the eutectic temperature when the excess volume parameters are fixed at −10^−6^, 0, and +10^−6^ m^3^ mol^−1^. It is evident that the eutectic point of the system with a negative interaction parameter for liquid is much lower than that of the ideal mixing alloy. Significantly, the direction of the eutectic temperature change is very negative when the excess volume parameter is negative, whereas the eutectic temperature is pushed to a higher value as the excess volume parameter becomes positive.

For the end-members (pure substance), the melting point decreases with decreasing particle size because the surface tension is the dominant factor and the surface tension of the solid is greater than that of the liquid (Equation (15)). The melting point of a pure substance generally increases with increasing pressure because the molar volume of the liquid is larger than that of the solid (Equation (20)). Of course, there are several exceptions when the molar volume of liquid is smaller than that of solid, so that the melting point decreases with increasing pressure (e.g., Bi [57]). The effect of pressure is closely related to the excess volume (Equation (23)). In this study, only the effect of excess volume of liquid is discussed, because the excess volume of solid is generally much smaller than that of liquid. When the excess volume of liquid is positive, the excess Gibbs energy increases. Accordingly, the eutectic temperature would be higher than that of a system having zero excess volume. Likely, when the excess volume of liquid is negative, the excess Gibbs energy decreases. Hence, the eutectic point moves to the lower temperature. 

**Figure 5:**
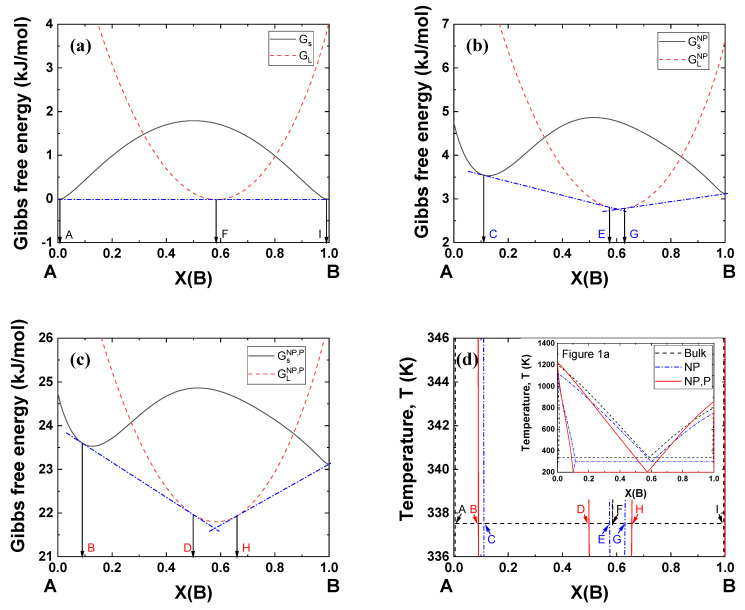
Gibbs energy curve of the A–B binary system at 338 K in Figure 1a: *V^Ex^* = −1 cm^3^/mol, Ω*_s_* = 15 kJ/mol, Ω*_L_* = −15 kJ/mol. (**a**) bulk, *P* = 0 GPa, (**b**) 5 nm *NP*, *P* = 0 GPa (**c**) 5 nm *NP*, *P* = 2 GPa. (**d**) Compositions of the equilibrium phases in Figure 1a.

Figure 5 shows typical examples of Gibbs energy curves at 338 K (bulk eutectic temperature under 0 GPa) for the A–B binary system in Figure 1a. Figure 5a shows that a single common tangent line can be drawn passing two contact points on the Gibbs energy of the solid (A and I) and one contact point on the Gibbs energy of the liquid (F, eutectic composition). When the particle size becomes 5 nm, the Gibbs energies of both the solid and liquid move upward, yielding two common tangent lines and a narrow liquid region (E~G) (Figure 5b). When a pressure of 2 GPa is applied to the nanoparticles, the Gibbs energies move to much higher positions, forming a wider liquid region (D–H) (Figure 5c). This simulation methodology can be used to identify nanoparticle systems which can potentially be used under high pressure.

## 4. Conclusions

The effects of size and pressure on the shape of the phase diagram of nanoparticles were examined using the CALPHAD method for general A–B alloy systems based on a regular solution model. The nanoparticle that was considered had a radius of 5 nm at pressures of 1 and 2 GPa. In order to examine the change in the eutectic point, the interaction parameter of the solid was taken to be +15 kJ mol^−1^, whereas that of the liquid was −15 or 0 kJ mol^−1^. The excess volume parameter for the solid was assumed to be zero ($V_{A,B\left(s\right)}=0$), whereas that for the liquid ($V_{A,B\left(l\right)}$) was taken as −10^−6^, 0, and +10^−6^ m^3^ mol^−1^. The following conclusions were obtained. 

(1) When the interaction parameter of the liquid was −15 kJ mol^−1^, the eutectic temperature decreased during the change from bulk to nanoparticles, and it further decreased when high pressure was applied to the system. The extent of the reduction in eutectic temperature became more considerable when the excess volume parameter was negative. For the change from bulk to nanoparticles, the eutectic composition moved in the direction of the low melting temperature element, B. When applying high pressure, the eutectic composition moved back in the direction of the high melting temperature element, A. 

(2) When the interaction parameter of the liquid was 0 kJ mol^−1^, the eutectic temperature decreased during the change from bulk to nanoparticles. It also decreased when high pressure was applied to the system when the liquid had a negative excess volume, whereas it increased when the liquid had a positive excess volume. The observed change in direction of the eutectic temperature is similar to the results obtained when the interaction parameter of the liquid was −15 kJ mol^−1^.

The present results can indicate a direction for the synthesis of nanoparticles under high pressure and application of nanoparticles under extreme conditions such as high temperature and high pressure. 

## Data Availability

The data that support the findings of this study are available from the corresponding author upon reasonable request.
